# Application of Advanced Oxidation Technology in Sludge Conditioning and Dewatering: A Critical Review

**DOI:** 10.3390/ijerph19159287

**Published:** 2022-07-29

**Authors:** Jiahua Xia, Juan Ji, Zhiqiang Hu, Ting Rao, Ankang Liu, Jingqian Ma, Yongjun Sun

**Affiliations:** 1 Nanjing Jiangbei New Area Public Utilities Holding Group Co., Ltd., Nanjing 210044, China; xiayu_610@sina.com (J.X.); jj13814186775@163.com (J.J.); airhzq@126.com (Z.H.); raot@mail.ustc.edu.cn (T.R.); 2 Nanjing Water Purification Environmental Research Institute Co., Ltd., Nanjing 211100, China; lakyx2@126.com; 3 College of Urban Construction, Nanjing Tech University, Nanjing 211800, China; majingqian123123@126.com

**Keywords:** advanced oxidation technology, sludge reduction, sludge conditioning, sludge dewatering, oxidation

## Abstract

Sludge dewatering is an important link in sludge treatment. In practical engineering, the dewatering effect of unconditioned sludge is very poor. The use of advanced oxidation technology can improve sludge dewatering performance, reduce sludge capacity, and remove micro-pollutants, which is beneficial for sludge post-treatment and disposal. Based on the current status of sludge conditioning and dehydration, the characteristics of the advanced oxidation method for sludge dehydration were systematically explained using various free radical reaction mechanisms and dehydration conditions. The effects of various advanced oxidation technologies on sludge conditioning and dewatering has been extensively discussed. Finally, the application prospects of the advanced oxidation technology in sludge conditioning and dewatering are presented.

## 1. Introduction

Considerable achievements have been made in China’s sewage treatment industry through the completion and operation of a large number of urban sewage treatment plants. The sharp increase in the amount of sewage treatment plants has led to the production of a large amount of activated sludge, which has led to a gradual increase in the load on sewage treatment plants [1]. Sludge treatment has become a key challenge for the Chinese water industry [2]. Sludge has the characteristics of high-water content, large volume, and high treatment cost, showing the performance of the fluid or semi-fluid state, causing great inconvenience for subsequent transportation and treatment [3]. 

Research focus. The main methods of sludge treatment are sanitary landfills and incineration. The moisture content of the sludge be less than 60% and 50%, respectively, based on the requirements of sanitary landfills and incineration [4]. In most cases, the moisture content of excess sludge is more than 95%, and there are four main types of sludge water, namely free water, interstitial water, adsorbed water, and bound water. After the excess sludge is concentrated, the moisture content is slightly reduced but still cannot meet the requirements of sanitary landfill and incineration [5]. Hence, further deep dewatering is required. Deep dewatering helps to protect the environment, strengthen land use, reasonably degrade a large amount of sludge pollutants, and ensure the safe integration of sludge resources into the circular ecosystem [6].

The methods of sludge conditioning include physical conditioning, chemical conditioning, and biological conditioning [7]. In physical conditioning, the microbial cells in the sludge are destroyed by physical methods, the structure of the sludge is changed, the binding effect of the sludge and water is reduced, and the inner water is released [8]. Among them, thermal conditioning has obvious effects on improving the dewatering rate of most sludges, but the treatment effect on sludges with low organic content is poor. Although microwave conditioning time is short, requires low heating temperature, has high thermal efficiency, and uses simple equipment, it requires a certain amount of sludge [9]. Freeze conditioning can be achieved spontaneously without adding the chemical additives, and the cost is low, but the scope of use is limited [10]. Ultrasonic conditioning has a high level of efficiency and environmental protection, and has application prospects, but its principle of action and complex effects are still to be discussed further [11]. Overall, the high energy consumption and high equipment cost of physical conditioning limit its large-scale use [12]. Microbial conditioning promotes mutual coagulation and precipitation of colloidal suspensions in water and improves sludge dewatering performance [13]. Microbial conditioning has strong advantages, such as high efficiency, non-toxicity, no secondary pollution, biodegradation, compact sludge floc, and a wide range of application [14]. However, the current research on such flocculants is not comprehensive, the research level is low, the mechanism of flocculants is not fully understood, and there are problems, such as large dosage, high production costs and low relevance [15].

Chemical conditioning is widely used as the optimal choice in the context of the comprehensive comparison. Advanced oxidation (AOP) is a typical chemical regulation method, and the free radicals it produces have strong oxidative effects [16]. Free radicals are generated by different oxidants, and different oxidants generate different free radicals with different reaction mechanisms [17]. Extracellular polymer (EPS) is the main component of sludge, and its components can promote the flocculation of sludge. However, too high an EPS content would lead to deterioration of the dehydration results due to hydrophilicity and strong intermolecular forces [18]. Therefore, reducing the EPS content and increasing its hydrophobicity is beneficial to the removal of water. Under the oxidation of AOP, EPS is effectively hydrolyzed, which weakens the strong binding ability of EPS and release the bound water in the sludge, thereby improving the dewatering performance of the sludge [19]. Advanced oxidation is an efficient sludge reduction treatment technology with characteristics of strong oxidizing ability, enhanced sludge-water separation effect, and it is not easy to generate secondary pollution [20]. Its application in the field of sludge treatment has gradually developed into a research focus in recent years.

This paper focuses on the application of different oxidation methods in sludge dewatering improvement and analyzes their applications in sludge dewatering and micropollutant removal. We have found that the advanced oxidation method destroys the EPS by free radicals, breaks the complex bond between water molecules and organic matter, reduces the hydration energy, dissolves the microbial cell wall, and helps the degradation of sludge and the removal of micro-pollutants in the sludge system. Hydroxyl and sulfate ions are more beneficial for improving the dewatering performance of sludge. However, before choosing the oxidizing agent and the AOP method, geographical conditions, purpose of use, level of economic development, and climatic conditions should be considered. This review could provide researchers with new ideas to employ multiple approaches for advanced oxidation by generating different or identical free radicals, thereby making it possible to develop new methods of dehydration that are more efficient, economical, and environmentally friendly.

## 2. Application of Hydrogen Peroxide in Sludge Dewatering

**Figure 1:**
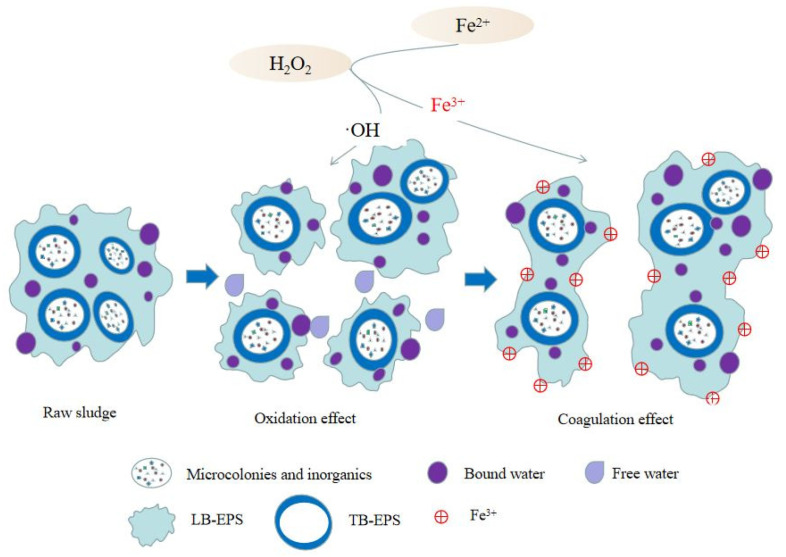
Mechanism of Enhanced Sludge Dewatering by Hydrogen Peroxide Pretreatment.

As a common oxidizing agent, hydrogen peroxide is widely used in advanced oxidation processes in Figure 1. The strongly oxidizing hydroxyl radicals formed in hydrogen peroxide play an important role in water treatment. Hydroxyl radicals inactivate bacteria during disinfection and release water through oxidation of EPS during sludge dewatering. Hydroxyl free radicals can also damage the cell wall of microorganisms or bacteria, increase the permeability of the cell membrane, destroy the cell wall, and release the intracellular water from the cell [21]. The Fenton system refers to a mixed system composed of Fe^2+^ and H_2_O_2_ [22]. Under acidic conditions, Fe^2+^ acts as a catalyst to promote the decomposition of H_2_O_2_ into hydroxyl groups with high reactivity. If Fe^3+^ is present, a Fenton-like process takes place at the same time, in which Fe^2+^ ions are formed in the Fenton process [23]. Fe^3+^ also reacts with water in solution and generating Fe^2+^ and hydroxyl radicals [24]. In the generation of ·OH by Fenton or Fenton-like methods, Fe^2+^ plays a key role in the initiation and progression of the reaction.

H_2_SO_4_ was used to adjust the pH of the sludge. Under the optimal dosage of Fe^2+^ and H_2_O_2,_ the SRF and CST of the sludge were reduced to 6.149 × 10^9^ m/kg and 15.7 s, which were 93% and 48.5% lower than the original sludge, respectively, indicating that the sludge dewatering effect is significantly improved [25]. LIU is also in the optimal dose. The sludge specific resistance SRF decreases by 95% below pH = 5, w(Fe^2+^) = 40 mg/g DS, w(H_2_O_2_) = 32 mg/g DS, lime = 400 mg/g DS [26]. Fenton’s reagent has been shown to be an effective strong oxidant in several experiments, but it also has several shortcomings and limitations. The pH of most Fenton reactions is below 4, which increases the burden on subsequent sludge treatment. In order to overcome this difficulty, the dewatering performance of sludge was tested under five systems, namely, Fenton-lime, Fenton, Fe^3+^, Fe^2+^, and H_2_O_2_, and it was found that the Fenton-lime, Fenton, Fe^3+^ system could significantly reduce the sludge SRF and CST [27]. Among them, Fenton and lime together adjusted the sludge, which can increase the pH while maintaining a good dehydration effect. Tao et al. further studied the effect of combining iron-rich biochar with H_2_O_2_ on sludge dewatering ability [28]. The iron contained in biochar combines with H_2_O_2_ to accelerate the Fenton reaction, and red mud is added as a framework material during the reaction. The WC of the mud cake decreased from 96.49% to 46.38%, and the CST and SRF decreased by 90.24% and 98.01%, respectively. The addition of biomass charcoal increases the drainage channel, which promotes the outflow of more sludge water under the same pressure during the dehydration process, thereby improving the dehydration efficiency.

Under the coupling action of Fenton, light, and ultrasonic waves, the polysaccharides in the sludge filtrate increased significantly, the particle size of the sludge became smaller than that under the action of Fenton oxidation alone, and the dewatering performance was significantly enhanced. Fenton oxidation coupled bioleaching with can improve sludge dewatering performance and remove trace metals in sludge [27,28,29]. For example, when the dosage of bioleaching 5d and H_2_O_2_ was 2 g/L, the removal effect of each micro-metal was significantly improved, and the removal rate of Cd increased from 90% to 99.5%, the removal rate of Cu and Zn increased from 60% to 70%, and Pb removal rate increased from 20% to 39% [29].

As shown in Table 1, the use of Fenton-type Fenton oxidation is fast, economical, and environmentally friendly, and the sludge dewatering performance and stability are significantly improved. The coexistence of Fe^2+^ and H_2_O_2_ can have an oxidizing effect. In addition, Fe^3+^ can also improve the dehydration performance. However, Fe^3+^ reacts with hydroxyl to form hydroxide precipitate, which increases the alkalinity of the solution and has a certain influence on the precipitation of Fe^3+^. Meanwhile, the generation of ·OH will be hindered if the H_2_O_2_ is excessive [30]. Therefore, researchers further strengthen sludge dewatering through Fenton coupling technology, which aids in the complete mineralization of organic matter, removes harmful substances such as trace metals in sludge, and reduces the environmental hazard of sludge. The Fenton reagent is coupled with the skeleton construct composite conditioner, which can form a rigid grid structure with high permeability in the mud cake, reduce the compressibility of the mud cake, and further enhance the dehydration effect. The Fenton reaction conditions are also acidic, and a neutralizing agent must *be* added to make the filtrate close to neutral. Yu used lime as the neutralizer, and through the corresponding surface design, the pH of the filtrate was increased under the premise of ensuring the dehydration effect [31]. Lime was used as the sludge floc skeleton, which can transfer part of the pressure to the inside of the floc under high pressure and provide a channel for water release [32]. However, lime production generates a considerable amount of CO_2_, and therefore red mud has been used as a neutralizer to cause the red mud to form a new mineralization stage and a hard network structure to help free water flow out [33]. In addition, slaked lime, pulverized coal, lignite, and sulfuric acid modified fly ash are also commonly used in the sludge conditioning process. Therefore, the combined use of Fenton reagent and physical conditioner can effectively improve the sludge drying rate and reduce the pH value of the filtrate, but issues, such as high treatment cost and low utilization rate of hydroxyl radicals, persist. Through the coupling of Fenton technology with light and ultrasonic waves, the cost of conditioners can be reduced effectively, the scope of application of sludge can be increased, and is more environment friendly as it ensures the dewatering effect.

## 3. Application of Persulfate in Sludge Dewatering

**Figure 2:**
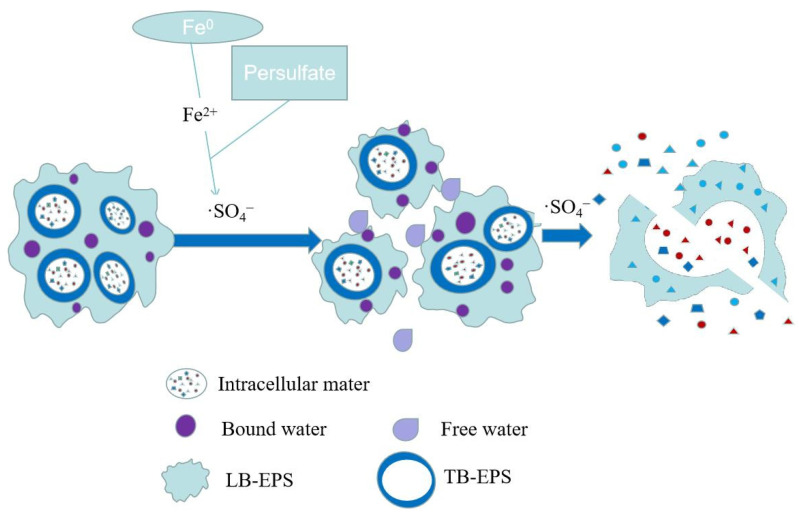
Mechanism of enhanced sludge dewatering by persulfate pretreatment.

As shown in Figure 2, compared with the advanced oxidation process of hydrogen peroxide, one of the advantages of the sulfate oxidation method is that the raw material is solid, which makes it easy to transport and store. Persulfate (PS) and peroxymonosulfate (PMS) are commonly used as sulfate-generating oxidants [35]. Persulfate can ionize persulfate ion (S_2_O_8_^2−^) in water. Its oxidizing ability is comparable to that of ozone, and its oxidation potential is 2.01 V. Under the catalytic activation of light, heat, transition metals, etc., a large amount of ·SO_4_^−^ can be generated, and the oxidation potential is up to 2.60 V. The advanced sulfate oxidation process also has other excellent advantages, such as longer half-life and wider operating pH range (2–10).

Zhen et al. used activated persulfate to enhance sludge dewatering for the first time and reported that at room temperature, persulfate in the pH range of 3.0–8.5 was catalytically activated by transition metal ions (Fe^2+^) and acted on excess sludge, which could significantly improve the sludge dewatering performance [36]. In addition, when the S_2_O_8_^2−^ and Fe^2+^ were 1.2 mmol/g (calculated by the dosage in 1 g of volatile suspended matter VSS, the same below) and 1.5 mmol/g, the CST decreased rapidly, which could be reduced by 88.8% within 1 min. The ·SO_4_^−^ produced by Fe^2+^ catalytic activation has strong oxidizing property. ·SO_4_^−^ can destroy the floc structure of sludge, release EPS bound water and intracellular bound water, and greatly improve the dewatering performance of sludge. Zhou et al. used zero-valent iron instead of Fe^2+^ as S_2_O_8_^2−^ inducer and found that when K_2_S_2_O_8_ was 4 g/L and Fe^0^ was 15 g/L, the sludge CST was reduced by more than 50%. Heat treatment was introduced into the Fe^2+^/S_2_O_8_^2−^ pretreatment system and the iron-sulfur oxidation-heat coupling integrated sludge enhanced dewatering technology was constructed. Moreover, the disintegration of EPS and separation of sludge and water were strengthened through a synergistic mechanism, which effectively reduced the dosage of chemicals and saved costs. When the temperature was 80 °C, the Fe^2+^ dosage was 1.2 mmol/g, and the S_2_O_8_^2−^ dosage was 1.5 mmol/g, the CST decreased by 96.5% (88.8% at normal temperature), the structure of the sludge floc was destroyed, and the bound water of EPS was released. The sludge dewatering efficiency also improved greatly [37].

As shown in Table 2, different from Fenton oxidation, when activated persulfate is used to enhance sludge dewatering, pH adjustment is not required, and thus, the operation is simple, fast and efficient. It is an efficient and promising sludge pretreatment enhanced dewatering technology. The ·SO_4_^−^ produced by Fe^2+^/S_2_O_8_^2−^ can realize high-efficiency degradation of EPS, and under the coupling action of low-pressure electrolysis Fe^2+^/S_2_O_8_^2−^, the micellar structure of sludge bacteria and the microbial cell wall are dissolved and destroyed, resulting in a large amount of sludge and the releasing of floc interstitial water and intracellular bound water. However, the oxidation of S_2_O_8_^2−^ and Fe^2+^ and its derivative processes are still in the exploratory stage. The effect of using zero-valent iron to replace Fe^2+^ as a spontaneous catalyst is not ideal because Fe^0^ needs to dissolve and decompose Fe^2+^ first, and then induce S_2_O_8_^2−^ to form ·SO_4_^−^, and the surface of Fe^0^ is easily passivated, resulting in the failure of Fe^0^ in the inner core, and thus, the dehydration efficiency cannot be compared with that of Fe^0^. Fe^2+^ is comparable to [38]. In addition, the oxidation potential of persulfate is 2.01 V when applied to sludge dewatering, the reaction rate is too low, and the effect is not ideal. However, under the catalytic activation of heat, light, transition metals, etc., persulfate can generate a large amount of ·SO_4_^−^, and the oxidation potential is as high as 2.60 V. High heat and high-pressure pretreatment destroys the sludge floc structure and improves the sludge dewatering performance, but the heat treatment consumes considerable energy, and thus, the operating cost is high. Electrolysis is also an effective method for sludge conditioning and enhanced dewatering, and the sludge treatment by electrolysis has little effect on the environment. However, the results showed that although low-pressure electrolysis alone could improve the sludge dewatering efficiency, it had limited effect on the closely attached EPS. In the future, the sludge enhanced dewatering process and theoretical system based on Fe^2+^/S_2_O_8_^2−^ oxidation should be further enriched and improved to provide reserve technologies for the front-end safety treatment and end-end ecological disposal of sludge.

## 4. Application of Ozone in Sludge Dewatering

**Figure 3:**
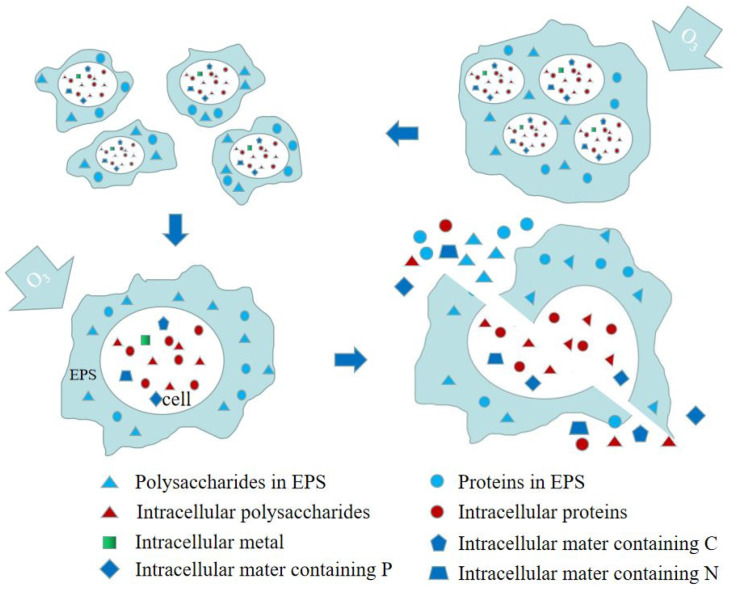
Enhanced sludge dewatering mechanism by ozone pretreatment.

As shown in Figure 3, the mechanism of action of O_3_ includes direct and indirect reactions. Ozone first destroys the sludge floc structure by oxidizing EPS and bridges and breaking up the colloidal structure. Since ozone has a strong oxidizing power, it damages cell membranes, releasing intracellular and extracellular substances into the liquid phase. Ozone can react with EPS and soluble substances in cells, including proteins, polysaccharides, and intracellular substances containing C, N, and P. Intracellular substances containing C can generate volatile fatty acids, resulting in a drop in pH. Ozone can also mineralize dissolved EPS and organic matter released by cells to generate CO_2_ and H_2_O, resulting in a reduction in the amount of ozone that reacts with the sludge. After ozonation, the mixed liquor suspension and mixed liquor volatile sludge suspended solid were reduced due to cell lysis. Authors should discuss the results and how they can be interpreted from the perspective of previous studies and of the working hypotheses. The findings and their implications should be discussed in as broad a context as possible. Future research directions can also be highlighted.

Chacana et al., treated the primary and digested sludge with ozone, and found that the SCOD and biodegradable COD of the primary sludge did not increase under the action of ozone oxidation; however, part of the organic matter was mineralized and converted to CO_2_ [44,45]. When the ozone mass concentration was 37.8 mg/g SS, the oxidation efficiency was the best, but the sludge CST increased significantly from (25.5 ± 2.4) s to (289.0 ± 25.9) s, and the filtration performance decreased significantly [46] because the size of the sludge flocs becomes smaller, and part of the free water is converted into adsorbed water. The polysaccharides and proteins in the filtrate increased from (4.46 ± 0.21) mg/L to (220.90 ± 24.87) mg/L and from (6.26 ± 0.28) mg/L to (386.54 ± 32.15) mg/L, respectively. The polysaccharides and proteins in EPS also move into the sludge when the intracellular polysaccharides and proteins are released. Therefore, polysaccharide and protein content increased while EPS decreased. Demir et al. also confirmed that after ozone treatment, the water content of the sludge cake was reduced and the sedimentation rate was accelerated, which could improve the mud-water separation efficiency to a certain extent, but the sludge SRF increased and the filtration performance decreased [45]. The degree of mineralization of sludge increased after O_3_ treatment, VSS/SS decreased from 0.86 to below 0.50, and 35% to 95% of solid carbon was mineralized. Weemaes reported that with an O_3_ dosage of 0.2 kg/kg COD, approx. 67% of the solid organic components were converted, 29% of which were dissolved, and the remaining 38% were directly mineralized [47]. After sludge ozonation, both inorganic and organic matter was dissolved into the supernatant, and the reduced value of MLVSS was slightly smaller than that of MLSS. The decrease in the MLVSS is the main reason for the decrease in the MLSS. With the increase in O_3_ dosage, the organic matter entering the supernatant becomes much larger than the inorganic matter, and thus, the sludge VSS/SS decreases with the increase in the degree of ozonation, thereby promoting the invisible growth of the sludge and reducing the sludge dry base production. The higher the soluble organic matter (SCOD), the more favorable for the subsequent aerobic/anaerobic digestion of the sludge. Thermal-chemical technology was used to treat sludge and it was found that SCOD increased significantly during O_3_ treatment [48]. At an O_3_ dose of 0.1 g/g DS, the SCOD increased from 240 mg/L to 960 mg/L. The increased SCOD can be attributed to O_3_ disrupting the microbial cell wall and releasing the cytoplasm into solution [49]. At the same time, the SCOD increase in the O_3_ strong oxidizing treatment solution of is used to ensure that the quality of the effluent during the O_3_ treatment process is up to the standard. The recirculation of ozonated sludge meant that the O_3_ treatment process did not affect the quality of the effluent. In this experiment, the SS and SCOD values of the effluent remained at 10 and 15 mg/L, respectively, while the dry sludge yield was greatly improved.

As shown in Table 3, the dewatering performance of sludge was significantly reduced after ozonation because as the size of the sludge flocs becomes smaller, part of the free water is converted into adsorbed water, the cells are ruptured, the intracellular substances and extracellular polymers enter the liquid phase, and the interstitial water and bound water content increased. When ozone and chitosan (CT) were combined for sludge conditions, effective sludge dewatering results were observed [50]. In the ozone/CT system, EP and sludge cells were destroyed by ozone, and CT as a coagulant could increase the size of sludge flocs and enhance the dewatering ability. Accordingly, rational control of the amount of O_3_ can stimulate its catalytic oxidation performance. At present, ozone is generally used as a catalytic material to catalyze the formation of ·OH from H_2_O_2_, which is used to strengthen sludge dissolution and methane fermentation. After ozonation, the hydrophobicity of sludge decreased, and the hydrophilicity increased. The effect of ozone on enhancing sludge dewatering is very limited, but ozone treatment promotes the anaerobic digestion process of sludge and improves methane yield. The anaerobic fermentation treatment of the excess sludge after ozone treatment can strengthen the anaerobic fermentation speed and increase biogas production, which has a significant effect on the energy conversion and reduction of sludge [51]. However, high ozone dose or long pretreatment time will lead to mineralization of dissolved organic matter and inactivation of key microorganisms, which is not conducive to methane production and energy recovery [52]. High cost is the main obstacle restricting the engineering application of ozone sludge treatment. In practice, factors such as sludge reduction and input cost should be considered comprehensively [53].

## 5. Application of Permanganate and Ferrate in Sludge Dewatering

**Figure 4:**
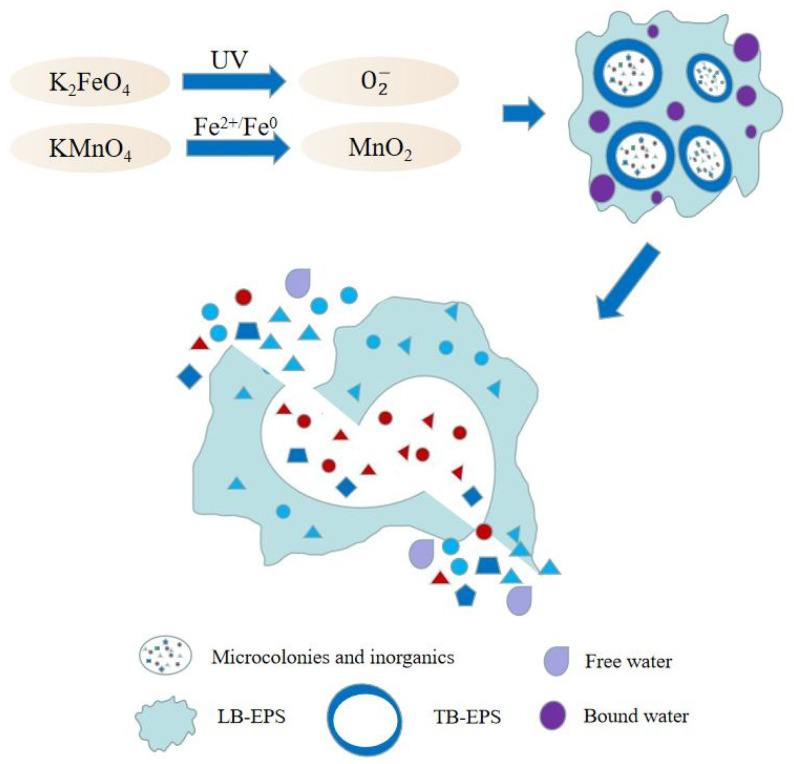
Mechanism of enhanced sludge dewatering by permanganate and ferrate pretreatment.

Recent research and applications have promoted the addition of H_2_O_2_ and PS/PMS as substrates that serve as catalysts for the production of hydroxyl and sulfate ions in Figure 4. In addition, potassium permanganate oxidation and ferrate oxidation can also be used as reliable oxidation methods in AOP. Under the catalysis of ferrous iron, permanganate can generate manganese dioxide with strong oxidation. Different forms of manganese dioxide can generate strong oxidizing·O_2_^−^ in the dark. Iron in ferrate is in an oxidized state and has a strong oxidizing property. At the same time, ferrate act as a flocculation when reduced to iron ions or ferric hydroxide.

Fe^2+^ and ZVI have catalytic effects on potassium permanganate, and under the catalysis of Fe^2+^, KMnO_4_ can react with it to generate strong oxidizing manganese dioxide (MnO_2_) [54]. After the sludge treatment, the WC, CST, and SRF of the sludge can be reduced effectively, and the effect is stronger than that of KMnO_4_ alone. The use of Fe(II)-KMnO_4_ has a highly effective effect on water purification, which includes the oxidation and flocculation ability of organic matter, and has been widely used in the treatment of high algae and arsenic-containing water. Likewise, heat or acid treatment prior to oxidation has also been proven effective, reducing the moisture content to 59.32%. It can be seen that permanganate itself has a certain oxidizing ability, and the catalytic system can improve the oxidizing ability of the permanganate. Under acidic and neutral conditions, the addition of ferrate can reduce WC in sludge without the aid of catalytic reagents. When using biochar as a framework material, the WC reduction is less than 60%. Therefore, the use of ferrate requires control of the appropriate pH for ferrate addition to work [55].

As shown in Table 4, the combined application of the potassium permanganate oxidation method can effectively improve sludge dewatering. The Fe(II)-KMnO_4_ system can be combined with pyrolysis sludge-based carbon (SBC) to construct a Fe-Mn-SBC system which aims to effectively adsorb AS(III) in groundwater and remediate groundwater pollution. Ferrate (VI) is also used in water treatment as a strong oxidant and disinfectant because iron is in the +6-oxidation state [56,57]. The oxidation mechanism is the strong oxidation of ferrate in the reduction process. The oxidizing power of ferrate (VI) is highly dependent on pH, water composition, type, and concentration of contaminants. Ferrate (VI) has a flocculation effect when reduced to iron ions or ferric hydroxide, and is an economical, energy-saving, and environmentally friendly chemical reagent, but maintaining a certain pH value will increase the use of acid/base and generate additional costs. In the latest study, Wu et al. found that when the ferrate (VI) system was formed using a combination of UV and ferrate (VI), the oxidative power increases and superoxide radicals could be generated. This result is more significant for the degradation of phenolic pollutants [58]. Through the coupling of ferrate and permanganate with other technologies, the cost of conditioners can be effectively reduced, the scope of application of sludge can be increased, and on the premise of ensuring the dewatering effect, is more environmentally friendly.

## 6. Application of Other Technologies in Sludge Dewatering

**Figure 5:**
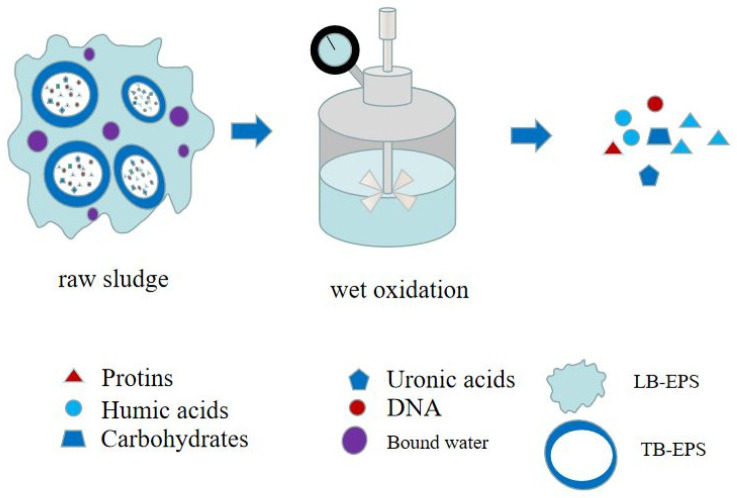
WAO pretreatment enhances sludge dewatering mechanism.

As shown in Figure 5, wet air oxidation (WAO) is a high-temperature and high-pressure oxidation treatment technology for organic or inorganic substances using oxygen as an oxidizing agent in a solution. Organic or inorganic substances can be converted into carbon dioxide, water, and biodegradable short-chain organic acids, with acetic acid being the most important short-chain organic acid. The influencing factors include reaction temperature, reaction pressure, pH value of the solution, reaction time, properties of the object to be treated, and the catalyst dosage. Heavy metals such as Cu and Ni, can be used as catalysts for wet air oxidation, namely catalytic wet air oxidation (CWAO). CWAO is developed based on WAO, with fast response speed and low running cost.

In wet air oxidation, insoluble organics substances are first converted into simple soluble organics by dissolution, and then further oxidized to carbon dioxide, water, and readily biodegradable intermediates [60,61]. The study found that the temperature in WAO had a significant influence on the dewatering performance of the sludge, and the dewatering performance was significantly improved at high temperature. In addition, the SCOD first increased and then decreased with increasing temperature, that is, the dewatering performance of the sludge was closely related to the soluble organic matter it contained. At a pressure of 24 MPa and under a constant amount of oxygen, the degradation rates of TSS and VSS increased from 74% and 85% to 85% and 92%, respectively, with a temperature increase from 220 °C to 240 °C, indicating an increase the temperature, thereby reducing the concentration of total/volatile suspended solids in the sludge [62]. High temperatures can promote the degradation of suspended solid SS [63]. Liang also confirmed that sludge dewatering performance is related to VSS% [32]. In addition to the reaction temperature, the oxygen content also has an influence on the sludge wet oxidation. The TSS/VSS value decreased with increasing oxygen content, indicating that changing experimental conditions affects the concentration of suspended matter. Therefore, to study the interaction between different influencing factors, the RSM method was used to analyze the effects of reaction temperature, oxygen dosage and reactor speed on the TSS, VSS and TCOD concentrations of the sludge at 5 and 60 min [64]. The three factors had a significant impact on the concentrations of TSS, VSS, and TCOD, and when the reaction reached 60 min, the rotation rate factor was *p* > 0. Throughout the course of the reaction, the reaction temperature is the main influencing factor. However, increasing the reaction temperature and oxygen dose promoted both the removal rate of TSS, VSS, and SS and the sludge yield.

A large number of studies have shown that WAO can be used directly in municipal sludge treatment, affecting sludge settling/dewatering performance, TSS and VSS content, TOC distribution, etc. Temperature and oxygen concentration have independent or synergistic effects on oxidation by affecting reaction kinetics or organic matter dissolution and gas-liquid mass transfer rates [65]. Organic carbon can be practically used as a denitrifying carbon source. Urrea studied the effects of reaction time, sludge characteristics and catalyst on TOC in the sludge supernatant, and found that the TOC concentration increased with the reaction time, and the TOC concentration was higher in the presence of the catalyst [61]. Since the supernatant contains a variety of organic carbons, especially acetic acid, Strong uses the supernatant as a denitrifying carbon source to obtain a good denitrification effect. However, WAO has the disadvantages of high energy consumption and high equipment investment. The WAO after adding the catalyst can speed up sludge conditioning and reduce the operating cost, and has a wide application background, but the catalyst of CWAO is heavy metal. CWAO faces challenges, such as catalyst separation and recovery and poisoning from by-products [66]. In the future, CWAO and WAO are planned be developed towards low cost, high stability, and easy recovery of toxic by-products.

## 7. Conclusions

This paper provides an overview of the application of various advanced oxidation technologies to improve sludge dewatering efficiency and demonstrates the applicability and efficiency of various advanced oxidation technologies to remove sludge contaminants. After treatment with Fenton’s reagent and activated persulfate, sludge dewatering performance and sludge-water separation efficiency improved significantly. Fenton, Fenton-like, and Fenton coupling technologies can not only enhance sludge dewatering but also effectively remove micro-metals in sludge to achieve sludge detoxification. Compared to ozone and H_2_O_2__,_ activated persulfate oxidation is more efficient in sludge dewatering. It can achieve rapid sludge dewatering in 1 min and persulfate is easy to store. Moreover, the generated ·SO_4_^−^ has a wider range of applications. It can play a role in the pH range and allow the remaining sludge to be treated at different pH levels. In contrast, ·OH is more beneficial in reducing SRF and sludge water content. It is worth noting that the oxidizing power of ·SO_4_^−^ is surpassed only to ·OH. At the same time, the external energy input significantly promoted the oxidation of free radicals and reduced the amount of the corresponding reagents that need to be added. The sludge treated with ozone had an improved sedimentation performance, which had an enhanced effect on further anaerobic fermentation. Both wet air oxidation and supercritical water oxidation were carried out at high temperature, high pressure and oxidant, and the organic matter in the sludge was directly oxidized into carbon dioxide, water, and intermediate products. The addition of permanganate and ferrate alone did not significantly improve the corresponding dehydration indices. Relevant catalytic means should be employed in these systems to exploit the potential level of free radical oxidation. There is a need to develop more conditioning and enhanced dewatering technologies for sludge dewatering, involving simple process, high efficiency, low consumption, environmentally friendly and low carbon. Therefore, optimizing the combination of advanced oxidation technology to achieve complementary advantages can maximize the advantages of advanced oxidation technology in sludge reduction applications.

## Figures and Tables

**Table 1:** Study results of enhanced sludge dewatering by hydrogen peroxide pretreatment.

Preprocessing Method	Optimal Pretreatment Conditions	Conditioning Dehydration Effect	Ref.
Fenton	Fe^2+^: 5000 mg/L; H_2_O_2_: 6000 mg/L	CST reduction: 48.5%; SRF reduction: 93.3%	[25]
Fenton	Fe^2+^: 25 mg/g DS; H_2_O_2_: 50 mg/g DS	CST reduction: 95.0%	[34]
Lime + Fenton	Fe^2+^: 40 mg/g DS; H_2_O_2_: 32 mg/g DS; pH: 5; Lime: 400 mg/g DS	SRF reduction: 95.0%	[26]
Lime + Fenton	Fe^2+^: 47.9 mg/g DS; H_2_O_2_: 34.3 mg/g DS; pH: 6.6 ± 0.2; Lime: 43.2 mg/g DS	Water content: 55.8 ± 0.6%	[27]
Lime + Fenton	Fe^2+^/H_2_O_2_: 50/30 mg/g DS; pH: 3; Lime: 50 mg/g DS; *t*: 10 min	SRF reduction: 90.0%	[27,32]
Red mud + Fenton	Fe^2+^: 31.9 mg/g DS; H_2_O_2_: 33.7 mg/g DS; Red mud: 275.1 mg/g DS	Water content: 59.8 ± 0.4%	[33]
Bioleaching + Fenton	H_2_O_2_: 2.0 g/L; *t:* 5 d	CST reduction: 89.8%; SRF reduction: 83.8%	[29]
Iron rich biochar + Fenton	Biochar: 0.792 g/g VS; H_2_O_2_: 0.072 g/g VS	CST reduction: 90.2%; SRF reduction: 98.0%; Water content: 46.0%	[28]

**Table 2:** Study results of enhanced sludge dewatering by persulfate pretreatment.

Preprocessing Method	Optimal Pretreatment Conditions	Conditioning Dehydration Effect	Ref.
Fe^2+^/S_2_O_8_^2−^	Fe^2+^: 1.5 mmol/g; S_2_O_8_^2−^: 1.2 mmol/g	CST reduction: 88.8%	[36]
Fe^2+^/S_2_O_8_^2−^	Fe^2+^: 1.5 mmol/g; S_2_O_8_^2−^: 1.2 mmol/g; T: 80 °C	CST reduction: 97.0%	[37]
Fe^0^/S_2_O_8_^2−^	Fe^0^: 15.0 g/L; S_2_O_8_^2−^: 4.0 g/L	CST reduction: 50.2%	[39]
Fe^0^/S_2_O_8_^2−^	Fe^0^: 2.0 g/L; S_2_O_8_^2−^: 0.5 g/L	CST reduction: 90.0%	[40]
Electrolysis + Fe^2+^/S_2_O_8_^2^^−^	Fe^2+^: 0.5 mmol/g; S_2_O_8_^2−^: 0.4 mmol/g;	Water content: 82.5%	[41]
Acid-ZVI/PMS	ZVI: 0.15 g/g DS; Oxone: 0.07 g/g DS; pH: 3	CST reduction: 19.6%; Water content: 76.3%	[42]
Fe^2+^/PMS	PMS 0.1 g/g TSS; Fe^2+^ 0.5 g/g TSS	CST reduction: 66.0%; SRF reduction: 88.0%	[43]

**Table 3:** Study results of enhanced sludge dewatering by ozone pretreatment.

Preprocessing Method	Optimal Pretreatment Conditions	Conditioning Dehydration Effect	Ref.
O_3_	O_3_: 37.8 mg/g SS	SS reduction: 13.5%;VSS reduction 11.9%	[46]
O_3_	O_3_: 0.5 g/g TS	SS reduction 77.8%; VSS reduction 71.6%	[45]
O_3_	O_3_: 0.1 g/g COD	Methane growth rate: 180%	[47]
O_3_	O_3_: 0.1 g/g DS	Methane growth rate: 25%	[49]
O_3_	O_3_: 50 mg/g DS	SS reduction: 49.1%; VSS reduction: 45.7%	[52]
O_3_ + CT	O_3_: 60 g/g TS; CT 20 mg/g TS	Water content: 56.5%	[29,50]

**Table 4:** Study results of enhanced sludge dewatering by permanganate and ferrate pretreatment.

Preprocessing Method	Optimal Pretreatment Conditions	Conditioning Dehydration Effect	Ref.
TA + Fe^0^/KMnO_4_	KMnO_4_ 0.4 mM/g DS; Fe^0^ 2.5 mMg/g DS; T: 45 °C	Water Content: 60.1%	[54]
Ferrate (VI)	VI: 25.3 mmol/L; T: 25 °C; pH: 7.0	tetrabromobisphenol A reduction: 99.1%	[57]
K_2_FeO_4_	K_2_FeO_4_: 1200 mg/L; pH: 3	SRF reduction: 30.5%; Water content: 73.4%	[55]
UV + Ferrate (VI)	VI: 0.1 g/L; pH: 7.0; UV: 0.198 mW/cm^2^	2,4-DCP degradation growth rate: 1020%	[58]
KMnO_4_ + Fe^2+^	KMnO_4_/Fe^2+^ = 3/1 (mass ration)	SRF reduction: 83.8%	[59]
